# Metalloproteases in Pain Generation and Persistence: A Possible Target?

**DOI:** 10.3390/biom13020268

**Published:** 2023-01-31

**Authors:** Gianmarco Marcianò, Cristina Vocca, Vincenzo Rania, Rita Citraro, Giovambattista De Sarro, Luca Gallelli

**Affiliations:** 1 Operative Unit of Pharmacology and Pharmacovigilance, “Mater Domini” University Hospital, 88100 Catanzaro, Italy; 2 Research Center FAS@UMG, Department of Health Science, University Magna Graecia, 88100 Catanzaro, Italy; 3 Medifarmagen SRL, Department of Health Science, “Mater Domini” University Hospital, 88100 Catanzaro, Italy

**Keywords:** MMPs, osteoarthritis, low back pain, neuropathic pain, MMP inhibitors

## Abstract

Matrix metalloproteinases (MMPs) are a large family of zinc-dependent proteolytic enzymes associated with extracellular matrix protein turnover and tissue degradation. They participate to many different physiological reactions but are also hyperactivated in several diseases. Various literature studies have documented that MMPs play a role in the modulation of neuropathic and nociceptive pain. The heterogeneity of clinical and pre-clinical data is an important issue in this experimental context. Despite the presence of a good number of studies on MMP inhibitors, these drugs showed scarce efficacy and relevant side effects. In the present manuscript, we reviewed studies in the literature that define a possible role of MMPs in pain and the effects of their modulation.

## 1. Introduction

**Figure 1:**
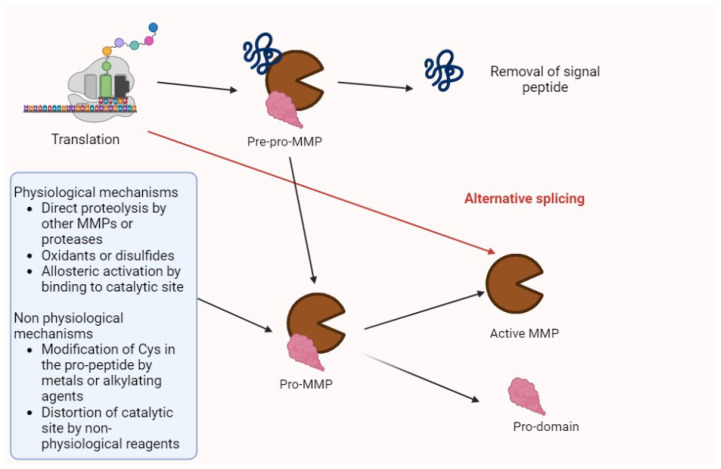
The activation of MMPs. After translation, MMPs are in the pre-pro-enzyme form, constituted by a signal peptide, a pro-domain, and enzyme complex. After the removal of signal peptides, the residual pro-MMP may be activated by several factors summarized in the figure. Then, the pro-domain undergoes proteolysis, and the active isoform may perform its biologic or pathologic functions. Alternative splicing may directly generate activated forms.

Matrix metalloproteinases (MMPs) are proteolytic enzymes involved in several processes (e.g., tissue regeneration, modifications of extracellular matrix (ECM), and the modulation of cytokines and chemokines). Their synthesis and activation is summarized in Figure 1 [1,2].

Pro-enzymes and the activated forms of MMPs are controlled by the tissue inhibitor of metallo-proteinases (TIMPs) and non-specific proteins (α2-macroglobulin and α1-proteinase inhibitors) [2].

To date, 24 MMP isoforms have been described in humans, with structural and functional differences (Table 1 and Table 2) [2,3].

**Figure 2:**
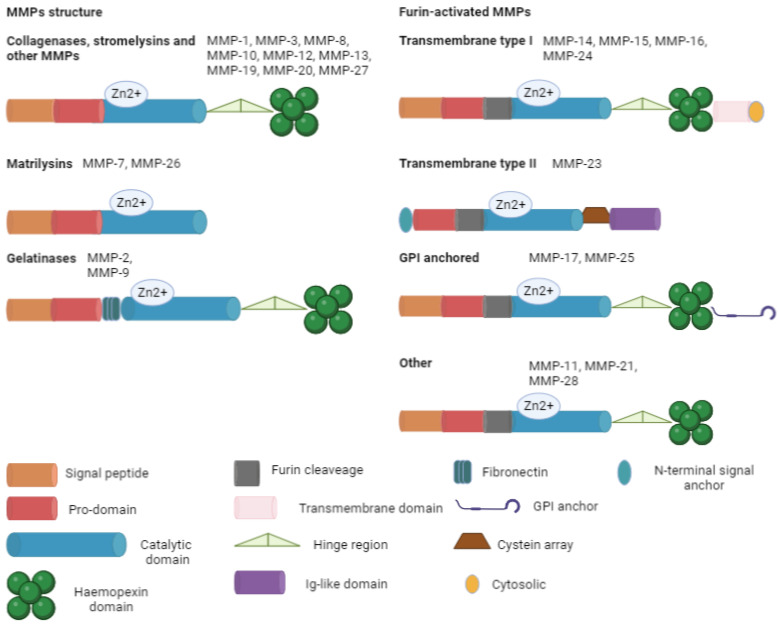
Main MMP structure. All the isoforms are basicly composed of a signal peptide (except for transmembrane type II), a pro-domain, and a catalytic domain. Furin-activated MMPs contain a furin recognition site near the catalytic domain, permitting the intracellular activation of the zymogen. GPI, glycophosphatidylinositol; Zn, zinc.

The ECM is made of various components: proteoglycans (syndecan-1 and aggrecan), fibers (fibronectin, elastin, collagen, and laminin), glycoproteins (tenascin, vitronectin, and entactin), and polysaccharides (hyaluronic acid). These substances regulate cell migration, growth, and differentiation. Collagen is the most important protein in the ECM, giving support to the structure of the cells. There are two main forms of collagen: fibrillar (types I, II, III, V, and XI) and non-fibrillar. The non-fibrillar type includes short chains (types VIII and X); facit-fibril-associated collagens with an interrupted triple helix (types IX, XII, XIV, XIX, and XXI); basement membranes (type IV); multi-plexin (types XV and XVIII); MACIT-membrane-associated collagens with an interrupted triple helix (types XIII and XVII); and other types (types VI, VII, and VIII). Collagen is composed of three chains in the triple helix, with two similar α chains (1 and 2). Degraded collagen is transformed in gelatin that can be further processed by MMPs. This plasticity of ECM has a relevant role in angiogenesis, wound healing, embryogenesis, morphogenesis, and tissue remodulation. The excessive degradation may lead to the development of pathologies (e.g., cancer, cardiovascular disease, immune disease, and metabolic disease) [2]. In this context, MMPs act as modulators. Their main constitutive elements are signal peptides with varying lengths that target the peptide for secretion; a pro-domain that maintains inactive MMPs and is then removed; a catalytic domain with a zinc ion; a link (the hinge region) that acts like a bridge between the catalytic domain and the hemopexin; a hemopexin domain; and an additional transmembrane domain. These components are variously expressed in the different isoforms, summarized in Figure 2.

MMPs play a role in inflammation and pain, regulating many functions including the activity and bioavailability of cytokines, growth factors, and chemokines. Furthermore, they play a role in tumor invasion, immune chemotaxis, and the regulation of inflammation in several disease [4,5,6,7,8,9,10,11,12,13,14,15,16,17,18,19,20].

However, cytokines also upregulate a wide variety MMPs (e.g., ADAMTS4 (disintegrin and metalloprotease with thrombospondin type I motifs), ADAMTS5, MMP-1, MMP-2, MMP-13, and MMP-14) which suppress the expression of essential ECM genes [21].

Cytokines, chemokines, adhesion molecules, and growth factors may interact with nociceptors. For instance, MMPs modulate these elements and then have an indirect influence [2]. Folgueras et al. [22] showed that MMP-24 deficiency led to enhanced sensitivity to thermal stimulation under basal conditions. This phenomenon was determined by increased innervation of the skin by mutant sensory neurons. After inflammation mice did not develop hyperalgesia.

The aim of this review is to describe and discuss the role of MMPs in pain. Moreover, we also assessed the possibility of a future MMP inhibition-based therapy.

## 2. Materials and Methods

The PubMed, Embase, and Cochrane library databases were searched for articles published until 10 December in agreement with our recent papers [23,24,25,26,27]. The secondary search included articles cited in the reference lists of papers identified by the primary search. The records were first screened by title/abstract (GM, VR) and then full-text articles were retrieved for an eligibility evaluation (CV). The remaining articles were then subject to a citation search of all reference lists (LG and GDS). Papers were deemed eligible if they included any of the words “MMPs”, “pain”, “TIMPs”, or “inhibitor”. All citations were downloaded into Mendeley, and duplicates were deleted. To avoid a bias of exclusion, the full-text articles were retrieved following the first-round exclusions and were also subject to two independent eligibility reviews, with perfect agreement this time. The studies evaluated as eligible were included in the present review. We excluded manuscripts without full text and without indications of effects on cancer, as well as manuscripts that were not in the English language.

## 3. MMPs in Pain

### 3.1. Acute Injury

Pain transmission, generation, and memory after acute trauma are complex phenomena inducing chronic pain, due to plastic changes in spinal cord (SC) with neuronal and glial alterations. In this process, the ECM plays a significant role.

Adams et al. [28], who studied the synovial fluid of 21 patients with acute ankle fracture and pain, documented significantly increased levels of MMP-1, MMP-2, MMP-3, MMP-9, and MMP-10 in an acute-phase study, suggesting a highly catabolic acute post-fracture environment, and increased levels of MMP-1, MMP-2, and MMP-3 6 months later. These data show the persistence of intra-articular inflammation (with an increase in MMPs and interleukins) after intra-articular ankle fracture healing, thus explaining the presence of pain in these patients and suggesting that a specific treatment must be performed in order to reduce inflammation, cytokines, and other inflammatory mediators [29].

In agreement with this study, in the same year (2015), Haller et al. [30], in a study on 45 patients with acute knee pain related to tibial plateau fractures, documented increased levels of MMP-1, MMP-3, MMP-9, MMP-10, and MMP-12. Two weeks later, the authors recorded an increase in MMP-13, suggesting that it requires a long time to increase, and its modulation is a delayed process. These data suggest that articular cartilage interacts with degradative proteases for a very long time following an acute injury; these proteases could play a role in the development of post-traumatic osteoarthritis, supporting the idea that an early treatment could reduce this expression.

**Figure 3:**
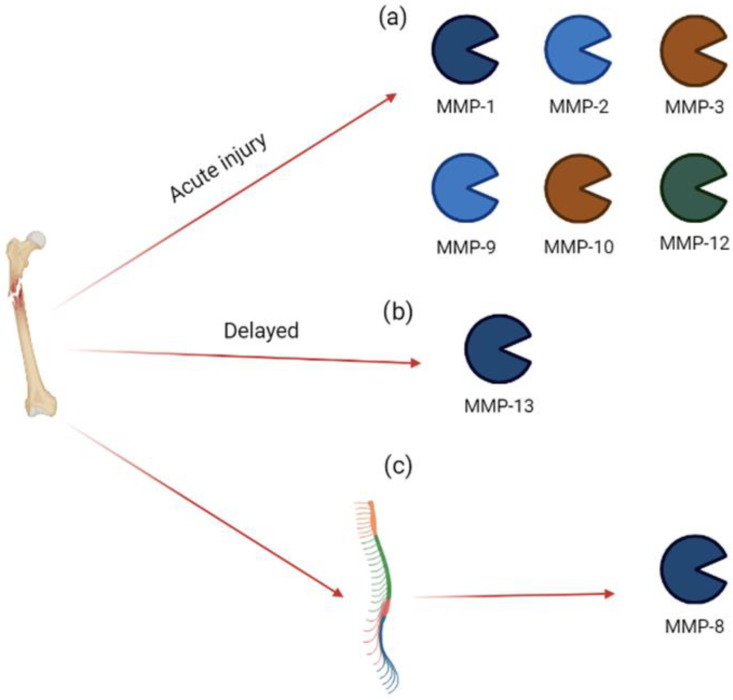
MMP expression in acute injury. Bone injury induces (**a**) an acute increase in MMP-1, MMP-2, MMP-3, MMP-9, MMP-10, and MMP-12 in synovial fluid; (**b**) a delayed increase in MMP-13 in synovial fluid; (**c**) an acute increase in MMP-8 in spinal cord.

In an experimental model of peripheral injury (tibia fracture), Tajerian and Clark [31] documented the development of acute severe pain and, three weeks later, an increase in MMP-8 expression in the spinal cord. In this model, the inhibition of MMP-8 using M8I (3R)-(+)-[2-(4-methoxybenzenesulfonyl)-1,2,3,4-tetrahydroisoquinoline-3-hydroxamate (1 mg/kg for 2 weeks) ameliorated the mechanical sensitivity. These data suggest that, following peripheral acute trauma, increased MMP-8 activity may alter ECM, generating a less rigid matrix without the ability of supporting structural changes in dendrites, leading to neuronal plasticity and chronic pain [31]. The involvement of MMPs in acute injury is summarized in Figure 3.

### 3.2. Osteoarthritis

Osteoarthritis is a chronic degenerative disease characterized by a degeneration of articular cartilage or subchondral bone with inflammatory cell activation, cytokine release, MMP activation, and chronic pain [32].

An in an vitro study [33], Kevorkian et al. found increased MMP-2, MMP-9, MMP-13, MMP-16, and MMP-28 levels, as well as reduced MMP-3 levels, in human cartilage from femoral heads.

The relationship between MMPs and the endocannabinoid system (ECS) has also been described in a previous experimental study by Dunn and et al. [34], demonstrating that synthetic cannabinoid agonists reduce MMP-3 and MMP-13 expression.

According to Galasso and colleagues’ literature review, MMP-1, MMP-3, MMP-7, MMP-8, and MMP-13 are involved in osteoarthritis pathogenesis, but the role of gelatinases (MMP-2 and MMP-9) is not negligible [35].

Pajak et al. [36] analyzed the expression of MMPs and the ECS in an experimental model of knee osteoarthritis, showing an increase in MMP-2, MMP-3, MMP-13, TIMP-1, and TIMP-2. The increase in MMPs (MMP-2 and MMP-13 in their active form) has been described at the beginning and the latter stages of pathologies, when higher levels of pain are present. The cartilage degradation is associated with higher pain perception, and it is related to the disequilibrium between TIMPs and MMPs. In fact, MMP3 expression is enhanced with respect to TIMP1 in synovial fluid.

Bahr and colleagues [37] assessed that the cannabinoid system is involved in several processes, such as pain regulation, head trauma, schizophrenia, multiple sclerosis, and seizures. The two main receptors are CB1 and CB2, i.e., two G-coupled receptors. In this context, CB1 is relevantly expressed in the central nervous system (CNS), but it is also present in the periphery. CB2 is considered a peripheral receptor; however, it has been found also in the CNS (glial cells and neurons). After the discovery of Δ9-tetrahydrocannabinol (THC) binding to this receptor, other endogenous ligands have been described, including anandamide and 2-arachidonyl glycerol. Other potential cannabinoids are noladin, virodhamine, and N-arachidonyldopamine. The increase in cannabinoids after pathologic events seems to indicate a compensatory system and CB1 is described as the main protagonist of these actions. The removal of these receptors in experimental models may increase susceptibility to several damage, including inflammation and seizures.

In osteoarthritis, higher expressions of cannabinoid receptors (CB1 and CB2) and anandamide (AEA) were reported to degrade enzymes. The increased expression of CB receptors reduces the pain, while the major degradation of AEA increases it [36].

**Figure 4:**
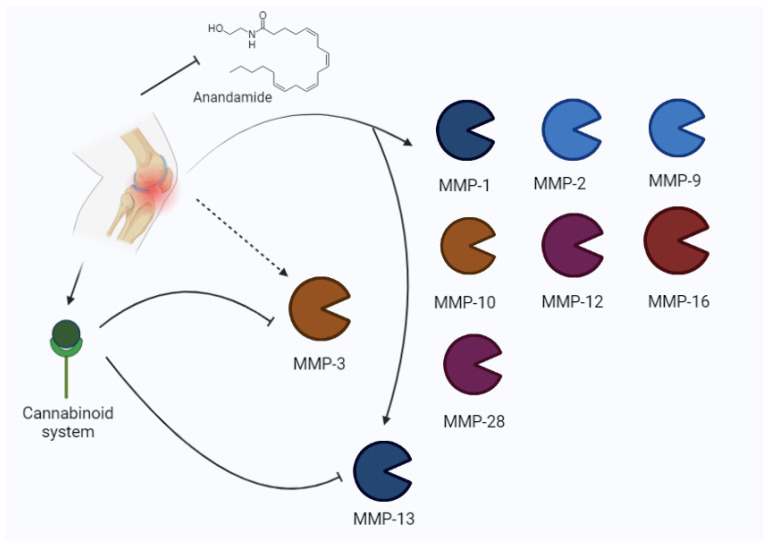
Osteoarthritis may induce an increase in MMPs (MMP-1, MMP-2, MMP-9, MMP-10, MMP-12, MMP-16, 28, MMP-3, MMP-13) and the modulation of the cannabinoid system. The cannabinoid system inhibits MMP-3 and MMP-13.

Mehana et al. [38] reported that MMP-13 is the most relevant isoform in osteoarthritis, according to its preferential action on type II collagen. It is one of the major factors involved in the degenerative process. Other important isoforms are MMP-1, MMP-2, MMP-3, and MMP-9. Moreover, MMP-1 and MMP-13 are rate-limiting in collagen degradation, whereas the other three isoforms are involved in non-collagen protein disruption [38]. Other authors described the role of MMP-13 in the progression of osteoarthritis [39,40,41], suggesting that MMP-13 is primarily involved in the development of pain in patients with OA. The role of MMPs in osteoarthritis is represented in Figure 4.

Furthermore, ADAMTS-12 seems to play a role in osteoarthritis. The ADAMTS-12 (activated by inflammatory cytokines) degradation of components such as the cartilage oligomeric matrix protein (COMP) results in the release of ECM constituents with pro-inflammatory activity. This pathway generates a feedback loop which feeds joint damage. Nevertheless, ADAMTS-12 also exhibits anti-inflammatory activity, in turn modulating pro- and anti-inflammatory cytokines [42,43].

### 3.3. Low Back Pain

The expression of MMPs and their role in low back pain have multiple implications.

The role of MMPs in the degeneration of intervertebral discs which can induce neck pain, vertebral pain, or low back pain is directly related to the overexpression of MMPs. In fact, the intervertebral disc consists of an outer fibrous ring, the annulus fibrosus (which is rich in collagen type I, providing strength), and the centrally located nucleus pulposus (which is rich in collagen type II and proteoglycans (mainly aggrecan)) [44]. Therefore, the overexpression of MMPs induces the degradation of collagens and proteoglycans with tissue degeneration and back pain.

Crean et al. [45] examined 34 discs from 29 patients with pain due to scoliosis and disc degeneration and observed higher expressions of MMP-2 and MMP-9. The authors showed that mechanical load may influence the expression of these enzymes.

Liou et al. [46] assessed the increase in MMP-2, MMP-9, MMP-17, and MMP-24 after sciatic nerve ligature and injury in animal models. In this paper, MMP-9 increased immediately after nerve injury, returning to normal levels on day 3, whereas MMP-2, MMP-17, and MMP-24 increased later.

Zhang et al. [47] observed the activity of MMPs in an animal model with spinal cord injury and documented the increased activity of MMP-2 and MMP-9 involved in the modulation of neuropathic pain and inflammation. Furthermore, MMP-9 has been associated with the chemotaxis of leukocytes, apoptosis, and the dissolution of the blood–spinal cord barrier. The most relevant achievement in this study is related to the different timings of MMP action. In fact, the early inhibition of MMPs improves symptoms, but MMP-2 plays a key role during wound healing and its inhibition is detrimental to the reparative process.

In an animal model of hyperalgesia induced by spinal cord injury, Miranpuri et al. [48] documented an increased expression of MMP-2, with respect to other groups without injury.

In agreement with these experimental studies, other authors documented a correlation between the MMP increase and clinical symptoms in patients with pain related to disc herniation.

In fact, in lumbar discs from patients undergoing lumbar spine fusion, a correlation between the increased expression of inflammatory cytokines, pain intensity, and disability has been described, showing that increased levels of MMPs induce increased levels of proinflammatory cytokines with the development of pain and disability [49].

Moreover, in disc degeneration, collagen (types I and II) is the principal target of destruction, and MMP-1 and MMP-13 are primarily involved in this degenerative process, even if other MMPs could be expressed [50].

In an experimental study, Kang et al. [51] evaluated 18 herniated cervical discs from 15 patients undergoing anterior disc surgery for pain and persistent radicular symptoms. The discs were analyzed, and biochemical findings revealed increased levels of MMPs (MMP-2, MMP-3, MMP-9, MMP-10, and MMP-11) and inflammatory mediators (IL-6 and prostaglandin E2) compared with the control discs. These data suggest that the MMPs of the group’s gelatinase and stromelysin (which degrades proteoglycan) are involved in the degenerative process of the disc and are also involved in the induction and maintenance of the inflammation, involved in clinical symptoms.

Bachmeier et al. [52] evaluated 37 discs from symptomatic disc herniation or degeneration and demonstrated the upregulation of MMP-3 and MMP-8, as well as of TIMP1 and TIMP2, suggesting that MMPs are enhanced in the degenerated disc and are synthesized by local cells, with an increase in TIMPs. These data support the previous results which explain the role of MMPs in the development of inflammation and disc degeneration that both induce pain and other symptoms.

Kameniak et al. [53] examined 70 subjects with disc herniation vs. 70 healthy patients at the time of admission, as well as 1 and 3 months after the surgery. The authors documented high TIMP-1 levels in patients with disc herniation vs. healthy patients 1 month after the surgery, which were correlated with a numerical rating scale for the back (NRS-B). In contrast, 3 months after surgery, they recorded higher levels of TIMP-2 with a reduced MMP-2/TIMP-2, and the ratio of patients with disc herniation vs. healthy subjects that were associated with an increase in the numerical rating scale for the leg (NRS-L).

More recently, Hsu et al. [54] analyzed both herniated disc tissue and the blood of 182 patients who underwent lumbar (93 subjects) or cervical (89 subjects) discectomy and showed that leptin induces IVD degeneration via the upregulation and activation of MMP-1.

Finally, in a study on 34 patients with low back pain, Aripaka et al. [55] documented a significant increased expression of MMPs (MMP-1, MMP-3, MMP-10, and MMP-13), ADAMTS (1 and 5), and proinflammatory cytokines (IL-6), thus suggesting their involvement in low back pain and in ECM metabolism, and that drugs that have the ability to inhibit these pathways could prevent a loss of ECM. An interesting point could be the association between MMPS expression, disc degeneration, and Modic-type changes. In fact, it has previously reported that disc degeneration is one factor leading to different types of Modic changes, supporting the idea that severe degeneration can lead to severe endplate damage, and hence type II changes often occur as well [56].

**Figure 5:**
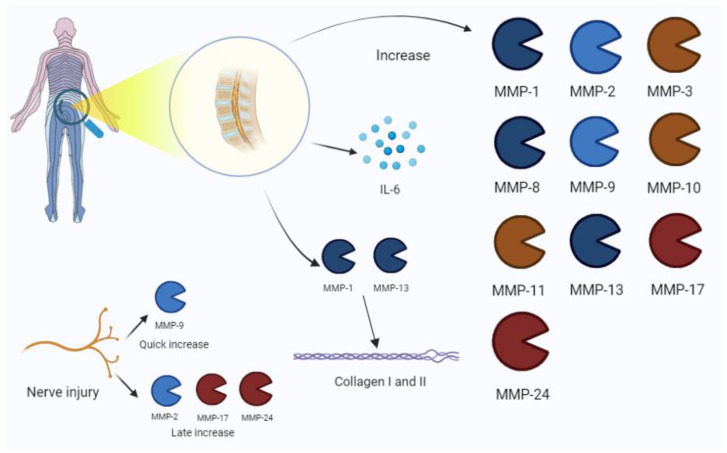
Low-back-pain-associated increase in MMPs. The process is also related to an IL-6 increase in disc pathology. Nerve injury is characterized by the expression of MMP-9 in early phases, and the late increase in MMP-2, -17, and -24. Among MMPs, MMP-1 and -13 are mainly involved in collagen disruption in intervertebral disc damage. IL, interleukin.

Interestingly, ADAMTS-7 and -12 were augmented in experimental models of disc degeneration, resulting in an increase in COMP fragments. These data suggest the role of these proteinases in IVD degeneration [42]. Figure 5 illustrates the main isoforms involved.

### 3.4. Neuropathic Pain

MMPs play a role in neuroinflammation’s ability to open the blood–brain barrier, cleave cytokines, and generate direct cellular damage, in turn killing neurons through their actions. MMP-2 and MMP-9 are involved in the development of neuropathic pain (increasing peripheral nerve injury), the mediation of pain hypersensitivity through IL-1β cleavage, and microglial–astrocytic activation [57,58]. The MMP-9/TIMP-1 axis is also implied in regulating Schwann cells in nerve repair [59].

According to Ahmed and colleagues [60], MMPs are relevantly involved in spinal cord injury. The balance between TIMPs and MMPs is significant in pain pathogenesis.

Cellular localization, the temporal pattern of MMP expression, and tissue distribution are the main factors that describe the relevance of their role. Mice with MMP-9 deficiency showed integrity disruption to the blood–spinal cord barrier (BSCB), reduced neutrophils, and infiltrated macrophages. Reduced tissue damage and quicker recovery were also observed. Nevertheless, MMP-9 also seems to have beneficial effects and MMPs are associated with the formation of glial scar, revascularization, and ECM remodeling (and with an increase in MMP-2) [60].

Spinal nerve injury allows us to understand the neuropathic pain mechanism and the involvement of MMPs. In the first few days following injury, MMP-9 upregulation in the dorsal root ganglion (DRG) becomes relevant, cleaving and activating IL-1β. Then, MMP-9 moves to dorsal horn, activating microglia through the action of IL-1β and p38 via the mitogen-activated protein kinase (MAPK). Successively (late-phase and maintenance response), MMP-2 increases in the DRG, inducing the activation of IL-1β and extracellular-regulated kinases (ERKs). MMP-2 is constitutionally expressed in healthy brain and spinal cord, causing MMP-9 levels to increase after injury [59].

Furthermore, MMPs may be responsible for pro-nerve growth factor (NGF) and pro-brain-derived neurotrophic factor (BDNF) activation [61]. However, other authors affirm that the NGF and the BDNF activate MMP-9 [62].

**Figure 6:**
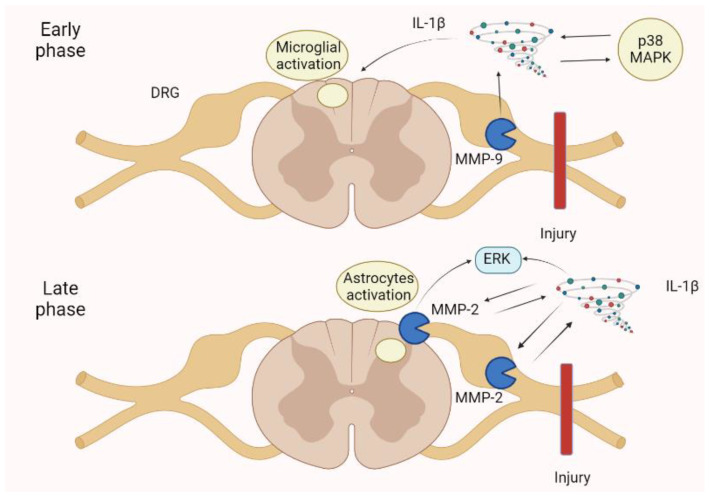
Neuropathic pain is spinal cord injury shows an early phase in which an MMP-9 increase in the DRG activates IL-1β, p38 MAPK, and then microglia. In the late phase, an MMP-2 increase in the DRG and spinal cord astrocytes determines a further enhancement of IL-1β and the ERK. These mechanisms augment pain perception and persistence, leading to neuropathic pain. ERK, extracellular-signal-regulated kinase; IL, interleukin; MAPK, mitogen-activated protein kinase.

Through experimental animal models, other authors have documented the role of MMP-9 in the development of neuropathic pain. In particular, Kawasaki et al. [58] documented that the administration of an IL-1β-neutralizing antibody reverts allodynia induced by MMP-9. Deng et al. [63] evaluated streptozotocin-induced diabetic neuropathy and highlighted the role of MMP-9 as an inductor of pathogenesis. These effects were reverted using alpha lipoic acid, a potent inhibitor of MMP-9. A schematic representation of MMPs actions in neuropathic pain is portrayed in Figure 6.

### 3.5. Orofacial Pain

According to orofacial pain’s main inflammatory origin, MMPs are estimated to play an important role in its pathogenesis. Tjaderhane et al. [64], by examining 37 caries samples after human teeth extraction, documented that MMP-2, MMP-8, and MMP-9, activated by bacterial acids, are involved in dentin disruption. High levels of MMP-8 and MMP-9 were documented in gingival crevicular fluid for advanced stages of periodontal disease [65,66,67,68]. Clinical conditions such as pulpitis show an increased expression of MMPs that is also documented in periapical lesions. Inflammatory cells may produce MMP-1 and MMP-3 [61].

In temporomandibular diseases (TMDs), the pathogenetic moment may be constituted by alterations of ECM components. For example, MMP-1, MMP-2, MMP-8, MMP-9, and MMP-13 were increased in the synovial fluid of patients with a derangement of temporomandibular joints (TMJs) [69,70]. Temporomandibular joints may be enriched by several inflammatory modulators, as testified by synovial fluid analysis. In this context, several proteinases and MMPs were increased [70]. Moreover, the MMPs of cartilage are estimated to be the main enzymes involved in the TMJ ECM breakdown [71]. Synovial inflammation is often associated with painful joint and synoviocites/macrophages through inflammatory cytokines and determines the release of pro-MMP3 in synovial fluid [61,72].

Moreover, MMP-9 seems to modulate satellite glial cells (SGCs), probably those related to pain persistence that strictly communicate with other nervous structures [73].

Interestingly, Nascimento et al. showed elevated levels of MMP-2 and MMP-9 in the trigeminal ganglion of experimental models. An MMP inhibitor reduced hyperalgesia and allodynia in rats. Enzymes produced in trigeminal ganglion may also move to the nerve’s periphery by extending the pathologic process [61,74].

**Figure 7:**
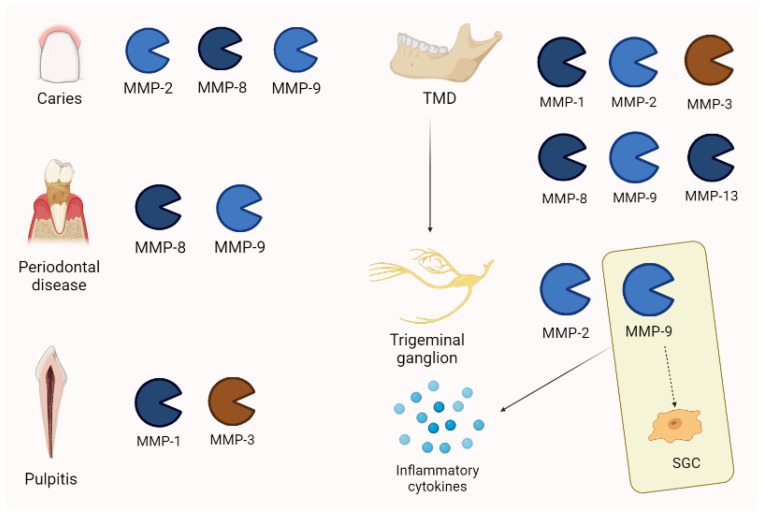
MMP expression in orofacial pain is different, depending on the pathology. Interestingly, in temporo-mandibular disease (TMD), MMP-2 and -9 are also increased in trigeminal ganglion. Moreover, MMP-9 is associated with pain persistency through its action on satellite glial cells (SGCs) and the activation of inflammatory cytokines.

Finally, MMP-2 and MMP-9 are involved in pain persistency with MMP-9 playing an active role in cytokines and the activation of pro-neurotrophins [61]. Main MMPs patterns are represented in Figure 7.

### 3.6. Tendinopathy

Tendinopathy is a generic term used to describe tendon pain without knowing the pathology. In particular, tendinitis is related to an inflammatory etiology, whereas tendinosis supposes a degenerative process with few or no inflammatory cells [75]. Rotator cuff is composed of four shoulder muscles: supraspinatus, teres minor, subscapularis, and infraspinatus. Rotator cuff pathology has various modalities and intensities, including injury, tendinopathy, partial tear, and complete tear. This clinical condition may determine severe pain and functional limitations [76].

Some interesting studies on rotator cuff tendinopathy reported an increase in MMP-13 levels [77,78]. In the torn rotator cuffs of 33 patients, Lakemeier et al. [79] observed an increase in the MMP-1 and MMP-9 levels and a decrease in MMP-3 levels. Jacob et al. confirmed that MMP-13 is overexpressed in 16 patients with rotator cuff tears and its levels were found to be related to pain severity [80].

Castagna et al. [81] analyzed 13 specimens from tendons of patients who underwent surgery for the repair of rotator’s cuff tendons. In both pathologic tendons samples and intact tendons, they observed an increase in MMP-1, MMP-2, MMP-3, TIMP-1, and TIMP-2. In samples of 10 patients with severe rotator cuff tear, Lo et al. [82] described an increase in MMP-13 and a decrease in MMP-3 mRNA. Furthermore, TIMP-2, -3, and -4 were reduced in the torn tendons.

Shih et al. [83], who measured the biomarkers of 42 subjects in the synovial fluid of a shoulder joint, observed higher levels of MMP-1 and MMP-13 in the massive full-thickness group compared to the others.

Tendinopathy, independently from localization, also correlates with MMP activity.

Magra et al. [84] reviewed the expression of MMPs in tendinopathy, showing that MMP-2 is upregulated in tendinopathy; it may be up- or downregulated in complete tendon tears and has the capacity of inhibiting TIMP-1/TIMP-2 in response to exercise. Even though MMP-1 is generally upregulated in tendon tears, tendinopathy, and shear stress, it is usually reduced in response to cyclical strain and static tensile load. For other MMP isoforms, MMP-13 is upregulated in complete tendon tears and MMP-9 after exercise. Evidence on TIMPs showed that TIMP-1 expression is briefly augmented (compensation) after acute tendon tear and reduced in tendinopathy, whereas TIMP-2, TIMP-3, and TIMP-4 are downregulated in both tendinopathy and tears.

**Figure 8:**
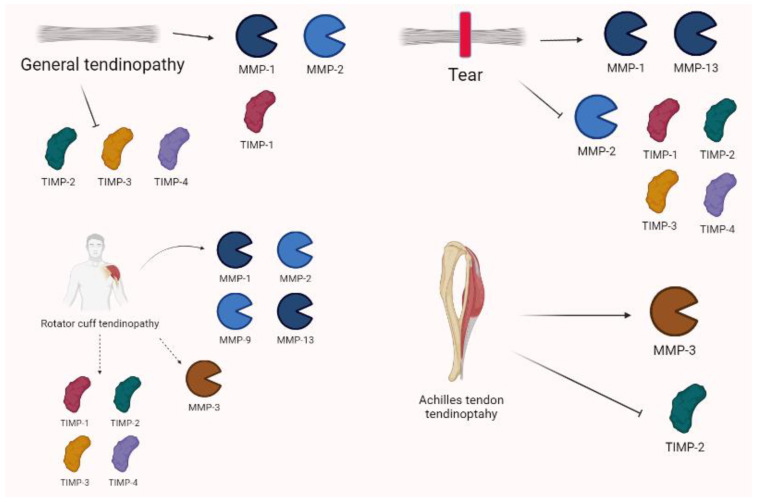
Tendinopathy shows complex and variable expressions of MMPs. In general, MMP-1 and -2 are often increased alongside TIMP-1. Conversely, TIMP-2, -3, and -4 are downregulated. Tears are characterized by increases in MMP-1 and MMP-13, as well as decreases in MMP-2 and TIMP-1, TIMP-2, TIMP-3, and TIMP-4. Specific settings show that rotator cuff tendinopathy variations are characterized by the overexpression of MMP-1, MMP-2, MMP-9, and MMP-13, whereas MMP-3 may be down- or upregulated. TIMP-1 is generally increased, whereas TIMP-2 may be both increased or decreased alongside TIMP-3 and -4 in tears. Achilles tendinopathy shows the relevant role of MMP-3 and TIMP-2 in pathogenesis.

According to Pasternak and Aspenberg, Achilles tendon rupture has a characteristic pattern of MMP expression/activity based on experimental models [85]. Moreover, Nie et al. [86], showed that two single nucleotide polymorphisms (SNPs), rs679620 (MMP3) and rs4789932 (TIMP-2), were significantly associated with chronic Achilles tendinopathy risk in 1084 patients. Godoy-Santos et al. [87] assessed the role of MMP-8 polymorphisms to determine the tendinopathy of the primary posterior tibial tendon and reported that it is involved in tissue destruction. A meta-analysis by Guo et al. [88] examined thirteen patients with 2871 cases of tendinopathy, and demonstrated that MMP-3 polymorphisms were considered relevant to determine illness and/or rupture. MMP-3 is commonly downregulated in tendinopathy, alongside TIMPs; this asset seems to contribute to clinical condition. In fact, MMP-3 seems to play an important role in the maintenance and modelling of the tendon. The expression of MMPs in tendinopathy is summarized in Figure 8.

### 3.7. Cutaneous Inflammation

**Figure 9:**
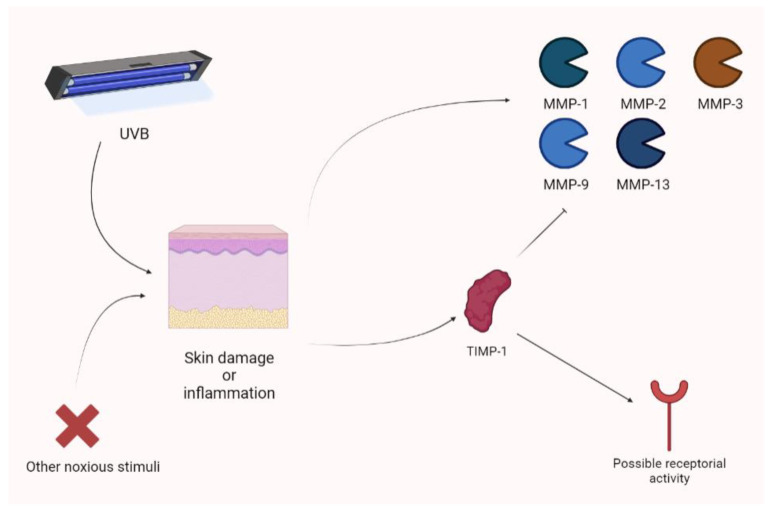
Cutaneous inflammation or damage is characterized by the raise of different MMP isoforms after UVB damage. Nevertheless, TIMP-1 seems to be overexpressed and to act as a regulator, not only thorough MMP inhibition, but also showing receptor-based activity. UVB, ultraviolet B.

Yokose and colleagues showed that UVB exposure photodamage leads to the upregulation of MMPs (MMP-1, MMP-2, MMP-3, MMP-9, and MMP-13). Their animal model, a xenograft of human skin, showed increased photoprotection when receiving a lentivirus vector expressing TIMP-1. The use of a TIMP-1-neutralizing antibody obtained the opposite result [89]. In an experimental model, Knight showed that cutaneous inflammation is associated with an increase in TIMP-1. In this context, TIMP-1 appears to not only act as an MMP inhibitor (the N-terminal domain), but also as a receptor ligand. Therefore, MMP-independent mechanisms are also considered, according to the analgesic action exerted by the TIMP-1 (C) domain. The increase in TIMP-1 is parallel to acute inflammatory pain and TIMP-1 knockout mice are characterized by hypersensitivity (persisting for long periods of time). Notably, the administration of recombinant TIMP-1 attenuated this phenomenon. Interestingly, an increase in TIMP-1 is associated with pain control only when its levels are significantly augmented in comparison to previous values [90]. Keratinocytes may upregulate TIMP-1 during inflammation. In fact, they produce TIMP-1 to attenuate photodamage [90] (Figure 9).

### 3.8. Rheumatology

**Figure 10:**
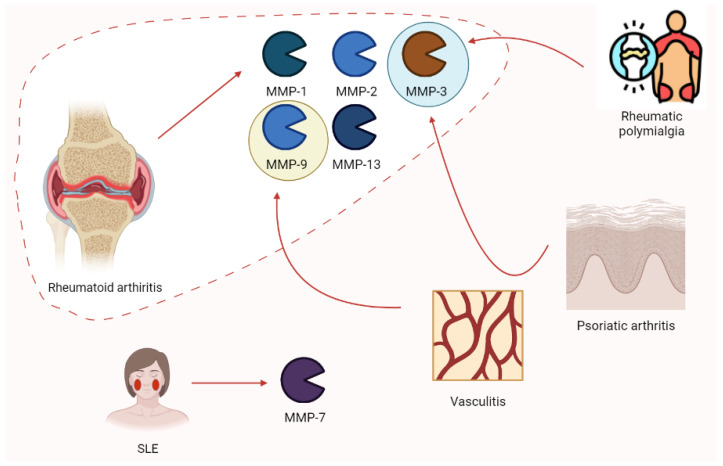
The expression of some of the main MMP isoforms in rheumatologic diseases is relevant to pathogenesis. Rheumatoid arthritis is characterized by the increases in MMP-1, -2, -3, -9, and -13. Nevertheless, other clinical conditions such as rheumatic polymyalgia, psoriatic arthritis, vasculitis, and SLE show this phenomenon. SLE, systemic lupus erythematosus.

Rheumatology has always been a hot spot for the study of MMPs. Ribbens et al. [91] highlighted the increase in MMP-3 in all rheumatologic conditions characterized by joint synovitis (RA, psoriatic arthritis, and polymyalgia rheumatica) after the collection of serum samples from 376 patients. Moreover, MMP-9 plays a role in vasculitis (e.g., Takayasu arteritis), possibly relating to clinical responses to pharmacologic treatment. Higher enzyme levels are associated with disease activity [92]. According to Vira and colleagues, MMP-7 levels increased in 150 SLE patients depending on clinical severity [93]. Rheumatoid arthritis (RA) is characterized by increases in MMP-2, MMP-3, and MMP-9. Moreover, MMP-1 and MMP-13 (proteoglycan) disrupt aggrecan and are involved in collagen degeneration. In this context, TIMPs are increased in synovia from RA patients with protective effects. Nevertheless, these patients are estimated to develop an autoimmune response to TIMPs [94]. Moreover, ADAMTS-12 seems to play a role in RA, according to the findings of COMP fragments in the cartilage and synovial fluid of patients with RA [42]. The role of MMPs in rheumatic pathologies is summarized in Figure 10.

## 4. Discussion

Metalloproteinase’s role in generating or facilitating pain insurgence has been widely described. Nevertheless, several points are still lacking sufficient evidence or practical solutions. Each MMP isoform has been addressed to a specific role in each pathology, leading to different phenomena. For example, the different properties and action timings of MMP-9 and MMP-2 in spinal cord pain are interesting aspects and are necessary to understand the role of MMPs and the possibility of developing new therapeutic strategies [59,63]). In fact, the inhibition of MMPs may be useful when determining pathologic actions, or deleterious when they are implied in physiologic reactions [95]. Some MMPs (e.g., MMP-10) have the power to activate other isoforms by extending the physiologic/pathologic process [55].

Another important point is related to MMPs playing their role, not as a single element, but as part of a very complex net made of genetics and environmental stimuli. These factors result in various cytokine expressions that regulate the production and activity of MMPs; TNF-α and IL-1β exert an important role in this process. For example, TNF-α induces MMP-2 through MMP-14 control. Moreover, IL-6 (induced by IL-1β and TNF-α) seems to potentiate TNF-α and IL-1β catabolic actions [55,83]. The transforming growth factor (TGF) β is activated by MMP-9 in fibroblast contraction models [96]. Nevertheless, TGFβ is also involved in the increased expression of MMP-9 in astrocytes and breast cancer cells [97,98].

The main problems relating to these data concern their provenience, mainly from experimental models and different pathological settings. The validity of some results must be confirmed in human clinical settings of a specific illness. Clinical research has tried to produce MMP-based treatments with different aims or modalities. This kind of approach has led to inconclusive results.

Paradoxically, Haro et al. [99] used intradiscal MMP-7 as a possible therapeutic option in herniated disc management. Their experimental model in vitro and in vivo showed a relevant decrease in the wet discs’ weight, reducing proteoglycan and water content. No damage in tissues around the disc was documented. Nevertheless, the differences between experimental models and human conditions, alongside the impossibility of assessing pain levels in animals, are important limitations.

Doxycycline, an antibiotic of the tetracyclines class, is an MMP inhibitor. Bedi et al. documented the improvement of rotator cuff tendons’ structure (after surgery) and MMP-13 inhibition by doxycycline in their experimental model. Tendon-to-bone repair was evaluated [95]. Pasternak and colleagues had a similar experience in Achilles tendon transection. However, their results were different to the previous study, with function worsening in animal models. They evaluated tendon-to-tendon repair, which may explain this different finding. Moreover, their evaluation was more functional than histological. Finally, doxycycline action on MMPs may also inhibit the physiological effects on the repair of these enzymes [100]. The experimental model by Khodir et al. highlighted that diclofenac and/or L-carnitine reduced knee swelling, inflammatory and oxidative stress markers, pain-related behaviors, and histopathological alterations in knee osteoarthritis. Moreover, they reduced MMP-13 and COX-2 expression. Diclofenac plus L-carnitine was better than the two agents alone [101].

According to Mahdavi et al., who studied 69 patients with knee osteoarthritis, L-carnitine significantly reduces MMP-1 and IL-1β [102]. Alpha lipoic acid is an inhibitor of MMP-9 (through action on NFκB) and MMP-2 and in vitro [103,104].

Moretti and colleagues [105] described the effect of intraarticular clodronate for the management of osteoarthritis. Clodronate is estimated to determine the apoptosis of macrophages and then to reduce the production of MMP-2, -3, and -9. The data from preclinical research and few clinical trials showed good responses for this treatment.

Corticosteroids are other well-known inhibitors of MMPs, with wide use in clinical settings in several diseases, even if their molecular role in MMP inhibition has not been fully understood [79]. Several MMP inhibitors have been used or tested in the management of cancer or other diseases. These molecules include synthetic peptides or non-peptidic molecules, bisphosphonates, tetracyclines, or natural inhibitors (e.g., TIMP-1). Despite their good efficacy in preclinical testing, these compounds (especially those not available on the market) only reached phase II in clinical trials. Musculoskeletal pain is often one of the most important concerns and adverse drug reactions [106]. Inhibitors of MMPs are considered potentially useful in several clinical settings. However, the biology and physiology of MMPs are not fully understood and the use of compounds acting without specificity can increase the risk of adverse drug reactions and clinical deteriorations. In fact, the inhibition of the physiologic action of MMPs may also cause relevant side effects (including gastrointestinal reactions). The main MMP inhibitor categories are summarized in Table 3.

Hydroxamates were the first compounds tested in clinical trials, showing good effectiveness in cancer animal models, even if their use is related to high toxicity and low efficacy [94,107]. This class acts through a molecular structure that mimics the natural substrate of MMPs and a group which chelates Zn^2+^ ions. The rest of the molecular backbone binds to the enzyme, with broad-spectrum inhibition and no specificity [94,107]. Non-hydroxamate compounds were created to overcome the chelation of metals of other metalloproteins and metabolic inactivation associated with the previous class. However, doxycicline is the only compound of this class approved for the management of periodontal disease [94,107]. Moreover, according to Serra and colleagues, who conducted a study on 64 patients, this drug may be also useful in treating chronic venous ulcers [19].

To avoid scarce selectivity on the specific MMP isoform, alternative sites of inhibition have been considered, leading to the development of new compounds. Other possible options are offered by antibody therapy and endogenous inhibitors. Antibodies may also target the activating MMPs to act indirectly on the pro-enzyme sited on the valley of the chain. A more detailed list of compounds is provided by Laronha et al. [94,107].

Research on selective compounds may be particularly useful in the management of osteoarthritis, as described by Wan and colleagues. The inhibition of MMP-13 may offer important symptomatic relief, blocking a relevant part of the pathogenetic process. This innovation would also be useful in reducing the use of other drugs commonly associated with adverse events (non-steroidal anti-inflammatory drugs, NSAIDs) [108].

According to Cabral-Pacheco et al., the use of TIMPs represents a new avenue in clinical research. In fact, TIMPs modulate MMPs, also promoting their activity. Nevertheless, their action (and that of MMPs) is not constant in each pathology and no progress has been achieved in this field [94]. Furthermore, the clinical efficacy of TIMPs has been observed in pre-clinical experience. Knight described improved symptoms in clinical inflammation and postulated the role of TIMP-1 as an alternative to opioid drugs. The antinociceptive mechanism of the protein may be affected by adverse drug reactions [90].

Another issue of relevance in most studies is the contrast between both transcriptional and protein activity levels. Increased mRNA concentrations do not correlate with a higher number of active proteins. A discrepancy between results and real-life effects may be related to this assumption. The microenvironment also plays a key role in MMP activity. For example, MMP-3 has an optimum pH window between 5.5 and 6.5 and is particularly active in disc degeneration where this condition is verified. Other MMPs may be less active in similar conditions, since each MMP possesses different pH optima [52].

It is not futile to stress the concept that MMPs may interfere with other known or unknown mechanisms. In the context of nerve sensitization, MMP-9 can mask opioid analgesia, without interfering with opioid-induced hyperalgesia. For example, the MMP-9 activation of IL-1β and its action on SGC may reduce opioid pharmacologic action [61]. Moreover, MMP-9-dependent glial fibrillary acidic protein (GFAP) induced by morphine subcutaneous injection increased in the satellite glial cells of DRGs [61].

Other complex molecular interactions may occur when drugs are administered. In cancer setting, opioids may modulate MMP-9 production and then cancer progression [109]. Morphine may determine this action through an independent opioid receptor, but the cell type is dependent on the mechanism. There are three main metabolic pathways in this process. Morphine chronic or high-dose use may activate adenylate cyclase and then cyclic adenosine monophosphate (cAMP), resulting in protein kinase A (PKA)/cAMP-responsive element-binding activation, and finally the inhibited production of the nuclear factor kappa light chain enhancer of activated B cells (NF-κB) and MMP-9 [109]. Acute or low doses can stimulate the opioid receptor, activating the Gβγ complex which, at the end of the chain, determines NF-κB activation and MMP-9 increases. The third additional mechanism is the increase in nitric oxide, resulting in NF-κB inhibition and MMP-9 decreases [109].

Last, it is crucial to remember the setting in which MMPs act. In fact, ECM should not be considered as a passive victim of their action, but as a main actor in the physiological and pathological events, leading to pain through various mechanisms [31] depending on the clinical setting.

## 5. Conclusions

In conclusion, we observed that MMPs are strongly involved in the pain generation, amplification, and persistence of several clinical conditions. They may be an intriguing therapeutic target, but the lack of specificity and the difficulty in targeting their pathologic activity explain the treatment failure in clinical trials. Several compounds have been produced, but they were not approved according to safety and efficacy concerns. Several elements, including the microenvironment, the clinical molecular context, the administration of other drugs, pH, the ECM, cytokine expression components, and other factors, are relevant to understand the net underlying the actions and reactions of MMPs. Pre-clinical and clinical data on all these factors must be segmented and collected for single pathology diagnostics, without making assumptions based on results relative to other conditions. Then, these achievements should translate to patients and unique characteristics in order to develop powerful compounds that can maintain the balance between hyperexpression and inhibition. This would be possible only through chemical analysis and engineering, resulting in more specificity and fewer side effects.

## Figures and Tables

**Table 1:** Matrix metalloprotease (MMP) structural classification.

Classification	MMPs
Non-furin-regulated MMPs	MMP-1, MMP-3, MMP-7, MMP-8, MMP-10, MMP-12, MMP-13, MMP-20, MMP-27
MMPs bearing three fibronectin-like inserts in the catalytic domain	MMP-2, MMP-9
MMPs anchored to the cellular membrane by a C-terminal glycosylphosphatidylinositol (GPI) Moiety	MMP-11, MMP-17, MMP-25
MMPs bearing a transmembrane domain	MMP-14, MMP-15, MMP-16, MMP-24
All the other MMPs	MMP-19, MMP-21, MMP-23, MMP-26, MMP-28

**Table 2:** MMP substrate specificity, domain organization, and sequential similarity classification [2].

Type of MMPs	Substrates
Collagenases	Types
MMP-1	Collagen types I, II, III, VII, VIII, X, and XI; entactin; tenascin; aggrecan; gelatin; fibronectin; vitronectin; myelin basic protein; ovostatin; and casein
MMP-8	Collagen types I, II, and III; fibronectin; aggrecan; and ovostatin
MMP-13	Collagen types I, II, III, IV, IX, X, and XIV; tenascin C isoform; fibronectin; laminin; osteonectin; casein; fibrillin-1; aggrecan core protein; gelatin; plasminogen; and serine proteinase inhibitors
MMP-18	Collagen and gelatin
Gelatinases	Types
MMP-2	Collagen types I, III, IV, V, VII, and X; gelatin; some glycoprotein of ECM; elastin; tenascin; fibronectin; laminin; aggrecan; myelin basic protein; and vitronectin
MMP-9	Collagen types IV, V, and XI; cytokines; entactin; myelin basic protein; casein; elastin; aggrecan; decorin; laminin; chemokines; IL-8; and IL-1
Matrylisin	Types
MMP-7	Faz ligand; pro-TNF-α; E-cadherin; syndecan-1; fibronectin; laminin; vitronectin; entactin; tenascin; elastin; casein; gelatin types I, II, IV, and V; collagen types I and IV; aggrecan; myelin; and proteoglycans
MMP-26	(in vitro): collagen type IV; fibronectin; fibrinogen; vitronectin; α1-antipripsin; β-casein; gelatin; α2-macroglobulin; and IGFBP-1
Membrane type	Types
MMP-14	Collagen types I, II, and III; gelatin; fibronectin; fibrilin-1; tenascin; entactin; aggrecan; laminin-1; vitronectin; cartilage proteoglycans; α1-proteinase inhibitor; and α2-macroglobulin
MMP-15	Laminin; fibronectin; gelatin; vibronectin; entactin; aggrecan; and tenascin
MMP-16	Gelatin; collagen type III; laminin; casein; and fibronectin
MMP-17	Gelatin; fibrinogen; and fibrin
MMP-24	Fibronectin; gelatin; and proteoglycans
MMP-25	Collagen type IV; fibronectin; gelatin; and proteoglycans
Stromelysin	Types
MMP-3	Collagen types I, II, III, IV, V, X, and IX; aggrecan; vitronectin; entactin; tenascin; fibronectin; gelatin; laminin; decorin; myelin basic protein; ovostatin; casein; osteonectin elastin; and proteoglycans
MMP-10	Collagen types III, IV, V, IX, and X; proteoglycans; gelatin; fibronectin; laminin; elastin; aggrecan; casein; and fibrilin-10
MMP-11	No protein of major relevance to ECM can be degraded by MMP-11, but it degrades the laminin receptor and serine proteinase inhibitors, α1-proteinases, and α1-antitrypsin inhibitors
Other MMPs	Types
MMP-12	Gelatin type I; elastin; laminin; vitronectin; proteoglycans; elastin; fibronectin; collagen types I, IV, and V; entactin; osteonectin; aggrecan; myelin; fibrinogen; and α1-antitripsin
MMP-19	Collagen types I and IV; laminin and nidogen; tenascin-C isoform; entactin; aggrecan; fibronectin; and gelatin type I (in vitro)
MMP-20	Ameloblasts; aggrecan; odontoblasts; and amelogenin
MMP-21	-
MMP-22	-
MMP-23	Gelatin
MMP-27	Gelatin
MMP-28	Casein

ECM, extracellular matrix; IGFBP, insulin-like growth factor binding protein-1; MMPs, matrix metalloproteinases.

**Table 3:** Main MMP inhibitor classes.

Classes	Compounds
Hydroxamate-based	Marimastat, ilomastat, batimastat New generation: cipemastat, prinomastat, MMI-270, MMI-166, PD-166793, ABT-770,
Non-hydroxamate	rebimastat, tanomastat, Ro 28-2653, doxycycline
Catalytic domain (non-zinc binding inhibitors)	PNU-141803, PNU-142372, neovastat, dieckol, and ageladine
Targeting alternative binding sites	NSC405020 and JNJ0966
Antibody-based therapies	REGA-3G12, REGA-2D9, AB0041, AB0046, andecaliximab (GS-5745), DX-2400, human scFv-Fc antibody E3, mAb 9E8, and LOOP_AB_
Endogenous inhibitors	TIMPs, α2-macroglobulin, tissue factor pathway inhibitor (TFPI), membrane-bound amyloid precursor protein, C-terminal proteinases enhancer protein, reversion-inducing cystein-rich protein with Kasal domain motifs (RECK), and GPI-anchored glycoprotein

## References

[1] Cui N., Hu M., Khalil R.A. (2017). Biochemical and Biological Attributes of Matrix Metalloproteinases. Prog. Mol. Biol. Transl. Sci..

[2] Laronha H., Caldeira J. (2020). Structure and Function of Human Matrix Metalloproteinases. Cells.

[3] Müller-Quernheim J. (2011). MMPs are regulatory enzymes in pathways of inflammatory disorders, tissue injury, malignancies and remodelling of the lung. Eur. Respir. J..

[4] Cione E., Piegari E., Gallelli G., Caroleo M.C., Lamirata E., Curcio F., Colosimo F., Cannataro R., Ielapi N., Colosimo M. (2020). Expression of MMP-2, MMP-9, and NGAL in tissue and serum of patients with vascular aneurysms and their modulation by statin treatment: A pilot study. Biomolecules.

[5] Serra R., Grande R., Buffone G., Gallelli L., De Franciscis S. (2013). The effects of minocycline on extracellular matrix in patients with chronic venous leg ulcers. Acta Phlebol..

[6] Serra R., Grande R., Montemurro R., Butrico L., Caliò F.G., Mastrangelo D., Scarcello E., Gallelli L., Buffone G., De Franciscis S. (2015). The role of matrix metalloproteinases and neutrophil gelatinase-associated lipocalin in central and peripheral arterial aneurysms. Surgery.

[7] de Franciscis S., Gallelli L., Amato B., Butrico L., Rossi A., Buffone G., Caliò F.G., De Caridi G., Grande R., Serra R. (2016). Plasma MMP and TIMP evaluation in patients with deep venous thrombosis: Could they have a predictive role in the development of post-thrombotic syndrome?. Int. Wound J..

[8] Serra R., Butrico L., Grande R., Placida G.D., Rubino P., Settimio U.F., Quarto G., Amato M., Furino E., Compagna R. (2015). Venous aneurysm complicating arteriovenous fistula access and matrix metalloproteinases. Open Med..

[9] Serra R., Volpentesta G., Gallelli L., Grande R., Buffone G., Lavano A., de Franciscis S. (2014). Metalloproteinase-9 and neutrophil gelatinase-associated lipocalin plasma and tissue levels evaluation in middle cerebral artery aneurysms. Br. J. Neurosurg..

[10] Serra R., Gallelli L., Butrico L., Buffone G., Caliò F.G., De Caridi G., Massara M., Barbetta A., Amato B., Labonia M. (2017). From varices to venous ulceration: The story of chronic venous disease described by metalloproteinases. Int. Wound J..

[11] Serra R., Gallelli L., Grande R., Amato B., De Caridi G., Sammarco G., Ferrari F., Butrico L., Gallo G., Rizzuto A. (2015). Hemorrhoids and matrix metalloproteinases: A multicenter study on the predictive role of biomarkers. Surgery.

[12] Serra R., Buffone G., Falcone D., Molinari V., Scaramuzzino M., Gallelli L., De Franciscis S. (2013). Chronic venous leg ulcers are associated with high levels of metalloproteinases-9 and neutrophil gelatinase-associated lipocalin. Wound Repair Regen..

[13] de Franciscis S., Gallelli L., Battaglia L., Molinari V., Montemurro R., Stillitano D.M., Buffone G., Serra R. (2015). Cilostazol prevents foot ulcers in diabetic patients with peripheral vascular disease. Int. Wound J..

[14] De Franciscis S., Mastroroberto P., Gallelli L., Buffone G., Montemurro R., Serra R. (2013). Increased plasma levels of metalloproteinase-9 and neutrophil gelatinase-associated lipocalin in a rare case of multiple artery aneurysm. Ann. Vasc. Surg..

[15] De Franciscis S., De Caridi G., Massara M., Spinelli F., Gallelli L., Buffone G., Caliò F.G., Butrico L., Grande R., Serra R. (2016). Biomarkers in post-reperfusion syndrome after acute lower limb ischaemia. Int. Wound J..

[16] Falcone D., Gallelli L., Di Virgilio A., Tucci L., Scaramuzzino M., Terracciano R., Pelaia G., Savino R. (2013). Effects of simvastatin and rosuvastatin on RAS protein, Matrix metalloproteinases and NF-κB in lung cancer and in normal pulmonary tissues. Cell Prolif..

[17] Gallelli L., Falcone D., Scaramuzzino M., Pelaia G., D’Agostino B., Mesuraca M., Terracciano R., Spaziano G., Maselli R., Navarra M. (2014). Effects of simvastatin on cell viability and proinflammatory pathways in lung adenocarcinoma cells exposed to hydrogen peroxide. BMC Pharmacol. Toxicol..

[18] Nissinen L., Kähäri V.-M. (2014). Matrix metalloproteinases in inflammation. Biochim. Biophys. Acta.

[19] Serra R., Gallelli L., Buffone G., Molinari V., Stillitano D.M., Palmieri C., De Franciscis S. (2015). Doxycycline speeds up healing of chronic venous ulcers. Int. Wound J..

[20] Serra R., Grande R., Gallelli L., Rende P., Scarcello E., Buffone G., Caliò F.G., Gasbarro V., Amato B., De Franciscis S. (2014). Carotid body paragangliomas and matrix metalloproteinases. Ann. Vasc. Surg..

[21] Rose K.W.J., Taye N., Karoulias S.Z., Hubmacher D. (2021). Regulation of ADAMTS Proteases. Front. Mol. Biosci..

[22] Folgueras A.R., Valdés-Sánchez T., Llano E., Menéndez L., Baamonde A., Denlinger B.L., Belmonte C., Juárez L., Lastra A., García-Suárez O. (2009). Metalloproteinase MT5-MMP is an essential modulator of neuro-immune interactions in thermal pain stimulation. Proc. Natl. Acad. Sci. USA.

[23] Pullano S.A., Marcianò G., Bianco M.G., Oliva G., Rania V., Vocca C., Cione E., De Sarro G., Gallelli L., Romeo P. (2022). FT-IR Analysis of Structural Changes in Ketoprofen Lysine Salt and KiOil Caused by a Pulsed Magnetic Field. Bioengineering.

[24] Di Mizio G., Marcianò G., Palleria C., Muraca L., Rania V., Roberti R., Spaziano G., Piscopo A., Ciconte V., Nunno N.D. (2021). Drug–Drug Interactions in Vestibular Diseases, Clinical Problems, and Medico-Legal Implications. Int. J. Environ. Res. Public Health.

[25] Muraca L., Scuteri A., Burdino E., Marcianò G., Rania V., Catarisano L., Casarella A., Cione E., Palleria C., Colosimo M. (2022). Effectiveness and Safety of a New Nutrient Fixed Combination Containing Pollen Extract plus Teupolioside, in the Management of LUTS in Patients with Benign Prostatic Hypertrophy: A Pilot Study. Life.

[26] Marcianò G., Roberti R., Palleria C., Mirra D., Rania V., Casarella A., Sarro G.D., Gallelli L. (2021). SARS-CoV-2 Treatment: Current Therapeutic Options and the Pursuit of Tailored Therapy. Appl. Sci..

[27] Chiarella G., Marcianò G., Viola P., Palleria C., Pisani D., Rania V., Casarella A., Astorina A., Scarpa A., Esposito M. (2021). Nutraceuticals for Peripheral Vestibular Pathology: Properties, Usefulness, Future Perspectives and Medico-Legal Aspects. Nutrients.

[28] Adams S.B., Setton L.A., Bell R.D., Easley M.E., Huebner J.L., Stabler T., Kraus V.B., Leimer E.M., Olson S.A., Nettles D.L. (2015). Inflammatory cytokines and matrix metalloproteinases in the synovial fluid after intra-articular ankle fracture. Foot Ankle Int..

[29] Gallelli L., Galasso O., Falcone D., Southworth S., Greco M., Ventura V., Romualdi P., Corigliano A., Terracciano R., Savino R. (2013). The effects of nonsteroidal anti-inflammatory drugs on clinical outcomes, synovial fluid cytokine concentration and signal transduction pathways in knee osteoarthritis. A randomized open label trial. Osteoarthr. Cartil..

[30] Haller J.M., Swearingen C.A., Partridge D., McFadden M., Thirunavukkarasu K., Higgins T.F. (2015). Intraarticular Matrix Metalloproteinases and Aggrecan Degradation Are Elevated After Articular Fracture. Clin. Orthop. Relat. Res..

[31] Tajerian M., Clark J.D. (2019). Spinal matrix metalloproteinase 8 regulates pain after peripheral trauma. J. Pain Res..

[32] Kucharz E.J., Szántó S., Ivanova Goycheva M., Petronijević M., Šimnovec K., Domżalski M., Gallelli L., Kamenov Z., Konstantynowicz J., Radunović G. (2019). Endorsement by Central European experts of the revised ESCEO algorithm for the management of knee osteoarthritis. Rheumatol. Int..

[33] Kevorkian L., Young D.A., Darrah C., Donell S.T., Shepstone L., Porter S., Brockbank S.M.V., Edwards D.R., Parker A.E., Clark I.M. (2004). Expression Profiling of Metalloproteinases and Their Inhibitors in Cartilage. Arthritis Rheum..

[34] Dunn S.L., Wilkinson J.M., Crawford A., Le Maitre C.L., Bunning R.A.D. (2014). Cannabinoid WIN-55,212-2 mesylate inhibits interleukin-1β induced matrix metalloproteinase and tissue inhibitor of matrix metalloproteinase expression in human chondrocytes. Osteoarthr. Cartil..

[35] Galasso O., Familiari F., Gori M.D., Gasparini G. (2012). Recent Findings on the Role of Gelatinases (Matrix Metalloproteinase-2 and -9) in Osteoarthritis. Adv. Orthop..

[36] Pajak A., Kostrzewa M., Malek N., Korostynski M., Starowicz K. (2017). Expression of matrix metalloproteinases and components of the endocannabinoid system in the knee joint are associated with biphasic pain progression in a rat model of osteoarthritis. J. Pain Res..

[37] Bahr B.A., Karanian D.A., Makanji S.S., Makriyannis A. (2006). Targeting the endocannabinoid system in treating brain disorders. Expert Opin. Investig. Drugs.

[38] Mehana E.-S.E., Khafaga A.F., El-Blehi S.S. (2019). The role of matrix metalloproteinases in osteoarthritis pathogenesis: An updated review. Life Sci..

[39] Wang M., Sampson E.R., Jin H., Li J., Ke Q.H., Im H.J., Chen D. (2013). MMP13 is a critical target gene during the progression of osteoarthritis. Arthritis Res. Ther..

[40] Chan C.M., Macdonald C.D., Litherland G.J., Wilkinson D.J., Skelton A., Europe-Finner G.N., Rowan A.D. (2017). Cytokine-induced MMP13 expression in human chondrocytes is dependent on activating transcription Factor 3 (ATF3) Regulation. J. Biol. Chem..

[41] Koskinen A., Vuolteenaho K., Nieminen R., Moilanen T., Moilanen E. (2011). Leptin enhances MMP-1, MMP-3 and MMP-13 production in human osteoarthritic cartilage and correlates with MMP-1 and MMP-3 in synovial fluid from OA patients. Clin. Exp. Rheumatol..

[42] Mohamedi Y., Fontanil T., Cal S., Cobo T., Obaya Á.J. (2021). ADAMTS-12: Functions and Challenges for a Complex Metalloprotease. Front. Mol. Biosci..

[43] Fontanil T., Mohamedi Y., Espina-casado J., Obaya Á.J., Cobo T., Cal S. (2021). Hyalectanase activities by the adamts metalloproteases. Int. J. Mol. Sci..

[44] Freemont A.J. (2009). The cellular pathobiology of the degenerate intervertebral disc and discogenic back pain. Rheumatology.

[45] Crean J.K., Roberts S., Jaffray D., Eisenstein S., Duance V. (1997). Matrix metalloproteinases in the human intervertebral disc: Role in disc degeneration and scoliosis. Spine.

[46] Liou J.T., Sum D.C.W., Liu F.C., Mao C.C., Lai Y.S., Day Y.J. (2013). Spatial and temporal analysis of nociception-related spinal cord matrix metalloproteinase expression in a murine neuropathic pain model. J. Chin. Med. Assoc..

[47] Zhang H., Chang M., Hansen C.N., Basso D.M., Noble-Haeusslein L.J. (2011). Role of Matrix Metalloproteinases and Therapeutic Benefits of Their Inhibition in Spinal Cord Injury. Neurotherapeutics.

[48] Miranpuri G.S., Schomberg D.T., Alrfaei B., King K.C., Rynearson B., Wesley V.S., Khan N., Obiakor K., Wesley U.V., Resnick D.K. (2016). Role of matrix metalloproteinases 2 in spinal cord injury-induced neuropathic pain. Ann. Neurosci..

[49] Aripaka S.S., Bech-Azeddine R., Jørgensen L.M., Chughtai S.A., Gaarde C., Bendix T., Mikkelsen J.D. (2021). Low back pain scores correlate with the cytokine mRNA level in lumbar disc biopsies: A study of inflammatory markers in patients undergoing lumbar spinal fusion. Eur. Spine J..

[50] Vincenti M.P., Brinckerhoff C.E. (2002). Transcriptional regulation of collagenase (MMP-1, MMP-13) genes in arthritis: Integration of complex signaling pathways for the recruitment of gene-specific transcription factors. Arthritis Res..

[51] Kang J.D., Georgescu H.I., McIntyre-Larkin L., Stefanovic-Racic M., Donaldson 3rd W.F.D., Evans C.H. (1996). Herniated cervical intervertebral discs spontaneously produce matrix metalloproteinases, nitric oxide, interleukin-6, and prostaglandin e2. Spine.

[52] Bachmeier B.E., Nerlich A., Mittermaier N., Weiler C., Lumenta C., Wuertz K., Boos N. (2009). Matrix metalloproteinase expression levels suggest distinct enzyme roles during lumbar disc herniation and degeneration. Eur. Spine J..

[53] Kamieniak P., Bielewicz J., Kurzepa J., Daniluk B., Kocot J., Trojanowski T. (2019). The impact of changes in serum levels of metalloproteinase-2 and metalloproteinase-9 on pain perception in patients with disc herniation before and after surgery. J. Pain Res..

[54] Hsu H.T., Yue C.T., Teng M.S., Tzeng I.S., Li T.C., Tai P.A., Huang K.F., Chen C.Y., Ko Y.L. (2020). Immuohistochemical score of matrix metalloproteinase-1 may indicate the severity of symptomatic cervical and lumbar disc degeneration. Spine J..

[55] Aripaka S.S., Bech-Azeddine R., Jørgensen L.M., Mikkelsen J.D. (2022). The expression of metalloproteinases in the lumbar disc correlates strongly with Pfirrmann MRI grades in lumbar spinal fusion patients. Brain Spine.

[56] Chen S., Huang Y., Zhou Z.-J., Hu Z.-J., Wang J.-Y., Xu W.-B., Fang X.-Q., Fan S.-W. (2014). Upregulation of tumor necrosis factor α and ADAMTS-5, but not ADAMTS-4, in human intervertebral cartilage endplate with modic changes. Spine.

[57] Kawasaki Y., Zhang L., Cheng J.K., Ji R.R. (2008). Cytokine mechanisms of central sensitization: Distinct and overlapping role of interleukin-1β, interleukin-6, and tumor necrosis factor-α in regulating synaptic and neuronal activity in the superficial spinal cord. J. Neurosci..

[58] Kawasaki Y., Xu Z.-Z., Wang X., Park J.Y., Zhuang Z.-Y., Tan P.-H., Gao Y.-J., Roy K., Corfas G., Lo E.H. (2008). Distinct roles of matrix metalloproteases in the early- and late-phase development of neuropathic pain. Nat. Med..

[59] Lakhan S.E., Avramut M. (2012). Matrix metalloproteinases in neuropathic pain and migraine: Friends, enemies, and therapeutic targets. Pain Res. Treat..

[60] Ahmed M.M., King K.C., Pearce S.M., Ramsey M.A., Miranpuri G.S., Resnick D.K. (2011). Novel targets for spinal cord injury related neuropathic pain. Ann. Neurosci..

[61] do Nascimento G.C., Leite-Panissi C.R.A. Matrix Metalloproteinases in Orofacial Pain: A Review. https://www.mathewsopenaccess.com/full-text/matrix-metalloproteinases-in-orofacial-pain-a-review.

[62] Dagnell C., Kemi C., Klominek J., Eriksson P., Sköld C.M., Eklund A., Grunewald J., Olgart Höglund C. (2007). Effects of neurotrophins on human bronchial smooth muscle cell migration and matrix metalloproteinase-9 secretion. Transl. Res..

[63] Deng X., Ma P., Wu M., Liao H., Song X.J. (2021). Role of matrix metalloproteinases in myelin abnormalities and mechanical allodynia in rodents with diabetic neuropathy. Aging Dis..

[64] Tjäderhane L., Larjava H., Sorsa T., Uitto V.J., Larmas M., Salo T. (1998). The activation and function of host matrix metalloproteinases in dentin matrix breakdown in caries lesions. J. Dent. Res..

[65] Kyrkanides S., O’Banion M.K., Subtelny J.D. (2000). Nonsteroidal anti-inflammatory drugs in orthodontic tooth movement: Metalloproteinase activity and collagen synthesis by endothelial cells. Am. J. Orthod. Dentofac. Orthop..

[66] Wahlgren J., Maisi P., Sorsa T., Sutinen M., Tervahartiala T., Pirilä E., Teronen O., Hietanen J., Tjäderhane L., Salo T. (2001). Expression and induction of collagenases (MMP-8 and -13) in plasma cells associated with bone-destructive lesions. J. Pathol..

[67] Avellan N.L., Kemppainen P., Tervahartiala T., Vilppola P., Forster C., Sorsa T. (2006). Capsaicin-induced local elevations in collagenase-2 (matrix metalloproteinase-8) levels in human gingival crevice fluid. J. Periodontal Res..

[68] Avellán N.L., Sorsa T., Tervahartiala T., Forster C., Kemppainen P. (2008). Experimental tooth pain elevates substance P and matrix metalloproteinase-8 levels in human gingival crevice fluid. Acta Odontol. Scand..

[69] Srinivas R., Sorsa T., Tjäderhane L., Niemi E., Raustia A., Pernu H., Teronen O., Salo T. (2001). Matrix metalloproteinases in mild and severe temporomandibular joint internal derangement synovial fluid. Oral Surg. Oral Med. Oral Pathol. Oral Radiol. Endodontol..

[70] Yoshida K., Takatsuka S., Hatada E., Nakamura H., Tanaka A., Ueki K., Nakagawa K., Okada Y., Yamamoto E., Fukuda R. (2006). Expression of matrix metalloproteinases and aggrecanase in the synovial fluids of patients with symptomatic temporomandibular disorders. Oral Surg. Oral Med. Oral Pathol. Oral Radiol. Endodontol..

[71] Planello A.C., Campos M.I.G., Meloto C.B., Secolin R., Rizatti-Barbosa C.M., Line S.R.P., De Souza A.P. (2011). Association of matrix metalloproteinase gene polymorphism with temporomandibular joint degeneration. Eur. J. Oral Sci..

[72] Ishimaru J.I., Oguma Y., Goss A.N. (2000). Matrix metalloproteinase and tissue inhibitor of metalloproteinase in serum and lavage synovial fluid of patients with temporomandibular joint disorders. Br. J. Oral Maxillofac. Surg..

[73] Chattopadhyay S., Shubayev V.I. (2009). MMP-9 controls Schwann cell proliferation and phenotypic remodeling via IGF-1 and ErbB receptor-mediated activation of MEK/ERK pathway. Glia.

[74] Nascimento G.C., Rizzi E., Gerlach R.F., Leite-Panissi C.R.A. (2013). Expression of MMP-2 and MMP-9 in the rat trigeminal ganglion during the development of temporomandibular joint inflammation. Braz. J. Med. Biol. Res..

[75] Charnoff J., Ponnarasu S., Naqvi U. Tendinosis. https://www.ncbi.nlm.nih.gov/books/NBK448174/#:~:text=Tendinopathy%20is%20an%20umbrella%20term,%20inflammatory%20response%20accompanies%20tendon%20injury..

[76] May T., Garmel G.M. Rotator Cuff Injury. https://www.ncbi.nlm.nih.gov/books/NBK547664/.

[77] Osawa T., Shinozaki T., Takagishi K. (2005). Multivariate analysis of biochemical markers in synovial fluid from the shoulder joint for diagnosis of rotator cuff tears. Rheumatol. Int..

[78] Shindle M.K., Chen C.C.T., Robertson C., DiTullio A.E., Paulus M.C., Clinton C.M., Cordasco F.A., Rodeo S.A., Warren R.F. (2011). Full-thickness supraspinatus tears are associated with more synovial inflammation and tissue degeneration than partial-thickness tears. J. Shoulder Elb. Surg..

[79] Lakemeier S., Braun J., Efe T., Foelsch C., Archontidou-Aprin E., Fuchs-Winkelmann S., Paletta J.R.J., Schofer M.D. (2011). Expression of matrix metalloproteinases 1, 3, and 9 in differing extents of tendon retraction in the torn rotator cuff. Knee Surg. Sport Traumatol. Arthrosc..

[80] Jacob J., Eisemon E., Sheibani-Rad S., Patel A., Jacob T., Choueka J. (2012). Matrix metalloproteinase levels as a marker for rotator cuff tears. Orthopedics.

[81] Castagna A., Cesari E., Garofalo R., Gigante A., Conti M., Markopoulos N., Maffulli N. (2013). Matrix metalloproteases and their inhibitors are altered in torn rotator cuff tendons, but also in the macroscopically and histologically intact portion of those tendons. Muscles Ligaments Tendons J..

[82] Lo I.K.Y., Marchuk L.L., Hollinshead R., Hart D.A., Frank C.B. (2004). Matrix metalloproteinase and tissue inhibitor of matrix metalloproteinase mRNA levels are specifically altered in torn rotator cuff tendons. Am. J. Sports Med..

[83] Shih C.A., Wu K.C., Shao C.J., Chern T.C., Su W.R., Wu P.T., Jou I.M. (2018). Synovial fluid biomarkers: Association with chronic rotator cuff tear severity and pain. J. Shoulder Elb. Surg..

[84] Magra M., Maffulli N. (2005). Matrix metalloproteases: A role in overuse tendinopathies. Br. J. Sports Med..

[85] Pasternak B., Aspenberg P. (2009). Metalloproteinases and their inhibitorsdiagnostic and therapeutic opportunities in orthopedics. Acta Orthop..

[86] Nie G., Wen X., Liang X., Zhao H., Li Y., Lu J. (2019). Additional evidence supports association of common genetic variants in MMP3 and TIMP2 with increased risk of chronic Achilles tendinopathy susceptibility. J. Sci. Med. Sport.

[87] Godoy-Santos A.L., Trevisan R., Fernandes T.D., dos Santos M.C.L.G. (2011). Association of MMP-8 polymorphisms with tendinopathy of the primary posterior tibial tendon: A pilot study. Clinics.

[88] Guo R., Aizezi A., Fan Y., Ji Z., Li W., Li Y., Wang Z., Ning K. (2022). Association between matrix metalloproteinase-3 gene polymorphisms and tendon-ligament injuries: Evidence from a meta-analysis. BMC Sports Sci. Med. Rehabil..

[89] Yokose U., Hachiya A., Sriwiriyanont P., Fujimura T., Visscher M.O., Kitzmiller W.J., Bello A., Tsuboi R., Kitahara T., Kobinger G.P. (2012). The endogenous protease inhibitor TIMP-1 mediates protection and recovery from Cutaneous Photodamage. J. Investig. Dermatol..

[90] Knight B. Role of Tissue Inhibitor of Matrix Metalloproteinase-1 in the Development of Hypersensitivity Using an Animal Model of Cutaneous Inflammation. https://opencommons.uconn.edu/dissertations/2324.

[91] Ribbens C., Martin y Porras M., Franchimont N., Kaiser M.J., Jaspar J.M., Damas P., Houssiau F.A., Malaise M.G. (2002). Increased matrix metalloproteinase-3 serum levels in rheumatic diseases: Relationship with synovitis and steroid treatment. Ann. Rheum. Dis..

[92] Serra R., Grande R., Buffone G., Scarcello E., Tripodi F., Rende P., Gallelli L., De Franciscis S. (2014). Effects of glucocorticoids and tumor necrosis factor-alpha inhibitors on both clinical and molecular parameters in patients with Takayasu arteritis. J. Pharmacol. Pharmacother..

[93] Vira H., Pradhan V., Umare V., Chaudhary A., Rajadhyksha A., Nadkar M., Ghosh K., Nadkarni A. (2017). Role of MMP-7 in the pathogenesis of systemic lupus erythematosus (SLE). Lupus.

[94] Cabral-Pacheco G.A., Garza-Veloz I., Rosa C.C.D.L., Ramirez-Acuña J.M., Perez-Romero B.A., Guerrero-Rodriguez J.F., Martinez-Avila N., Martinez-Fierro M.L. (2020). The roles of matrix metalloproteinases and their inhibitors in human diseases. Int. J. Mol. Sci..

[95] Bedi A., Fox A.J.S., Kovacevic D., Deng X.H., Warren R.F., Rodeo S.A. (2010). Doxycycline-mediated inhibition of matrix metalloproteinases improves healing after rotator cuff repair. Am. J. Sports Med..

[96] Kobayashi T., Kim H.J., Liu X., Sugiura H., Kohyama T., Fang Q., Wen F.Q., Abe S., Wang X., Atkinson J.J. (2014). Matrix metalloproteinase-9 activates TGF-β and stimulates fibroblast contraction of collagen gels. Am. J. Physiol.—Lung Cell. Mol. Physiol..

[97] Hsieh H.L., Wang H.H., Wu W.B., Chu P.J., Yang C.M. (2010). Transforming growth factor-β1 induces matrix metalloproteinase-9 and cell migration in astrocytes: Roles of ROS-dependent ERK- and JNK-NF-κB pathways. J. Neuroinflamm..

[98] Gomes L.R., Terra L.F., Wailemann R.A.M., Labriola L., Sogayar M.C. (2012). TGF-β1 modulates the homeostasis between MMPs and MMP inhibitors through p38 MAPK and ERK1/2 in highly invasive breast cancer cells. BMC Cancer.

[99] Haro H., Nishiga M., Ishii D., Shimomoto T., Kato T., Takenouchi O., Koyanagi S., Ohba T., Komori H. (2014). Experimental chemonucleolysis with recombinant human matrix metalloproteinase 7 in human herniated discs and dogs. Spine J..

[100] Pasternak B., Fellenius M., Aspenberg P. (2006). Doxycycline impairs tendon repair in rats. Acta Orthop. Belg..

[101] Khodir S.A., Al-Gholam M.A., Salem H.R. (2020). L-Carnitinepotentiatestheanti-inflammatoryandantinociceptive effects of diclofenac sodium in an experimentally-induced knee osteoarthritis rat model. Iran. J. Basic Med. Sci..

[102] Malek Mahdavi A., Mahdavi R., Kolahi S. (2016). Effects of l-Carnitine Supplementation on Serum Inflammatory Factors and Matrix Metalloproteinase Enzymes in Females with Knee Osteoarthritis: A Randomized, Double-Blind, Placebo-Controlled Pilot Study. J. Am. Coll. Nutr..

[103] Lee H.S., Na M.H., Kim W.K. (2010). α-Lipoic acid reduces matrix metalloproteinase activity in MDA-MB-231 human breast cancer cells. Nutr. Res..

[104] Kim H.S., Kim H.J., Park K.G., Kim Y.N., Kwon T.K., Park J.Y., Lee K.U., Kim J.G., Lee I.K. (2007). α-Lipoic acid inhibits matrix metalloproteinase-9 expression by inhibiting NF-κB transcriptional activity. Exp. Mol. Med..

[105] Moretti A., Paoletta M., Liguori S., Ilardi W., Snichelotto F., Toro G., Gimigliano F., Iolascon G. (2021). The rationale for the intra-articular administration of clodronate in osteoarthritis. Int. J. Mol. Sci..

[106] Nothnick W.B. (2004). Novel targets for the treatment of endometriosis. Expert Opin. Ther. Targets.

[107] Laronha H., Carpinteiro I., Portugal J., Azul A., Polido M., Petrova K.T., Salema-Oom M., Caldeira J. (2020). Challenges in matrix metalloproteinases inhibition. Biomolecules.

[108] Wan Y., Li W., Liao Z., Yan M., Chen X., Tang Z. (2020). Selective MMP-13 Inhibitors: Promising Agents for the Therapy of Osteoarthritis. Curr. Med. Chem..

[109] Khabbazi S., Hassanshahi M., Hassanshahi A., Peymanfar Y., Su Y.W., Xian C.J. (2019). Opioids and matrix metalloproteinases: The influence of morphine on MMP-9 production and cancer progression. Naunyn. Schmiedebergs. Arch. Pharmacol..

